# The Role of Distinct Subsets of Macrophages in the Pathogenesis of MS and the Impact of Different Therapeutic Agents on These Populations

**DOI:** 10.3389/fimmu.2021.667705

**Published:** 2021-08-20

**Authors:** Maedeh Radandish, Parvin Khalilian, Nafiseh Esmaeil

**Affiliations:** ^1^Department of Immunology, School of Medicine, Isfahan University of Medical Sciences, Isfahan, Iran; ^2^Environment Research Center, Research Institute for Primordial Prevention of Non-Communicable Disease, Isfahan University of Medical Sciences, Isfahan, Iran

**Keywords:** multiple sclerosis, macrophages, microglia, therapeutic agents, neuroinflammation

## Abstract

Multiple sclerosis (MS) is a demyelinating inflammatory disorder of the central nervous system (CNS). Besides the vital role of T cells, other immune cells, including B cells, innate immune cells, and macrophages (MФs), also play a critical role in MS pathogenesis. Tissue-resident MФs in the brain’s parenchyma, known as microglia and monocyte-derived MФs, enter into the CNS following alterations in CNS homeostasis that induce inflammatory responses in MS. Although the neuroprotective and anti-inflammatory actions of monocyte-derived MФs and resident MФs are required to maintain CNS tolerance, they can release inflammatory cytokines and reactivate primed T cells during neuroinflammation. In the CNS of MS patients, elevated myeloid cells and activated MФs have been found and associated with demyelination and axonal loss. Thus, according to the role of MФs in neuroinflammation, they have attracted attention as a therapeutic target. Also, due to their different origin, location, and turnover, other strategies may require to target the various myeloid cell populations. Here we review the role of distinct subsets of MФs in the pathogenesis of MS and different therapeutic agents that target these cells.

## Introduction

Multiple sclerosis (MS) is a demyelinating inflammatory disorder of the central nervous system (CNS). Neurodegeneration (loss of myelin and axons) in MS is caused by an immune response to self-antigens, interrupting signal transmission (1). MS patients exhibit various clinical symptoms related to the site of lesions and associated with the invasion of inflammatory cells across the blood–brain barrier (BBB). In most patients, the disease begins with a single episode, known as a clinically isolated syndrome (CIS), which might be developed in the future or not (2). Patients with at least two relapses are classified as relapsing-remitting multiple sclerosis (RRMS) that makes up >70% of the MS population. Primary progressive multiple sclerosis (PPMS) is another phenotype that occurs in approximately 10%–15% of individuals, and PPMS patients have no remission after the onset of disease (3, 4). Within 10–20 years after the disease onset, 60%–70% of RRMS patients develop secondary progressive MS (SPMS) symptoms by steady progression with or without periods of remission (5).

Although the cause of MS is unknown, genetic, epigenetic, and environmental factors have been introduced as the possible risk factors of the disease. Individuals with an inherited HLA-DRB1*15:01 allele and its associated haplotypes (DQB1*06:02, DQA1*01:02, DRB1*15:0, DRB5*01:01) are more likely to develop MS (6). Also, based on genome-wide association studies (GWAS), HLA locus has related with disease susceptibility in 20%–30% of MS patients (7), while some alleles are associated with resistance to MS. Accordingly, studies have found that HLA-DRB1*01:01, HLA-DRB1*09, HLA-DRB1*11, HLA-DRB1*12, and HLA-DRB1*16 alleles play a role in protection against MS (8, 9). Besides, other non-HLA genes such as interleukin (IL)-2RA, IL-7RA, CD58, signal transducer and activator of transcription (STAT)3, interferon regulator factor (IRF)8, and tumor necrosis factor receptor superfamily member 1A (TNFRSF1A) are involved in susceptibility to MS (10).

Environmental risk factors such as low vitamin D levels, smoking, obesity, stress, infections, and immunization have been considered as risk factors for MS development (11).

Experimental autoimmune encephalomyelitis (EAE) is an animal model for MS that is used in experimental studies. Many aspects of the MS pathophysiology, such as inflammation, immune surveillance, immune-mediated tissue injury, and roles of immune cells, have been revealed by using EAE models (12). Also, studies have shown that there is a correlation between EAE and MS therapeutic success. For example, licensed drugs such as disease-modifying therapies (DMTs), interferon (IFN)-beta, glatiramer acetate, and the anti-very late antigen (VLA)-4 antibody (natalizumab), have shown therapeutic efficacy in both MS and EAE (13–18). Therefore, EAE as an appropriate model has contributed to our scientific knowledge of neuroinflammation.

Besides the vital role of T cells, other immune cells, including B cells, innate immune cells, and macrophages (MФs), also play a critical role in MS pathogenesis (19, 20). MФs are innate immune phagocytes that detect pathogen-associated molecular pattern (PAMP) and damage-associated molecular pattern (DAMP) molecules. These molecules are expressed by pathogens and apoptotic cells, respectively. MФs also present antigens to T lymphocytes as an antigen-presenting cell (APC) in adaptive immunity. According to *in vitro* features, MФs are divided into M1 and M2 phenotypes. This nomenclature primarily represents the state of MФ’s activation and is used to facilitate the description of the inflammatory status; otherwise, their phenotype should be seen as plastic manner (21). *In vitro* exposure of monocytes and MФs to Th1 cytokines, lipopolysaccharide (LPS), and granulocyte-macrophage colony-stimulating factor (GM-CSF) induces their polarization to inflammatory M1 phenotype. These cells produce high levels of pro-inflammatory cytokines, including TNF-α, IL-6, IL-1β, and inducible nitric oxide synthase (iNOS) (22, 23). M1 MФs are the first line of defense against intracellular pathogens and control tumor growth. Also, M1 MФs probably play a role in tissue destruction and autoimmune disorders (24). On the contrary, *in vitro* differentiation of monocyte to M2 MФs is induced in the presence of Th2 cytokines and other immunomodulatory agents, including macrophage colony-stimulating factor (M-CSF), IL-10, transforming growth factor (TGF)-β, and vitamin D3 (25, 26). Recently, M2 MФs have been classified into four subgroups including M2a, M2b, M2c, and M2d. Generally, the M2 phenotype has anti-inflammatory characteristics and plays a role in the immune response against parasitic infections, allergic reactions, tissue regeneration, and tumor growth (27).

Recent studies have indicated that MФs possess distinct metabolic characteristics that correlate with their functional state, known as metabolic reprogramming. In the context of metabolic reprogramming, M1 MФs express iNOS enzyme to produce nitric oxide (NO) from arginine, present enhanced glycolytic metabolism, pentose phosphate pathway (PPP), fatty acid synthesis (FAS), and impaired Krebs [or tricarboxylic acid (TCA)] cycle and mitochondrial oxidative phosphorylation (OXPHOS). On the other hand, M2 MФs hydrolyze arginine to ornithine and urea by Arg-1 and are characterized by enhanced OXPHOS, FAS, glutamine metabolism, and decreased PPP. It is noteworthy that the different intracellular metabolic pathways regulate the polarization and function of M1 and M2 MФs (28–30). For example, in the M1 MФs, NO and NO-derived reactive nitrogen species inactivate the mitochondrial electron transport chain (ETC) and prevent repolarization to the M2 phenotype. On the contrary, ornithine can further participate in downstream pathways of polyamine and proline synthesis, which have a role in cell proliferation and tissue repair in M2 (31, 32). Also, based on studies, glycolysis may promote the immune function of M1 MФs by increasing the secretion of inflammatory cytokines and enhancing phagocytic activity (33).

Several subsets of MФs are present in the CNS. The resident MФs in the parenchyma are known as microglia. Also, non-parenchymal MФs are located in the choroid plexus, perivascular space, and meninges. These cells have a critical role in the maintenance of CNS homeostasis (34–36). The other types of MФs in the CNS are the monocyte-derived MФs entering the CNS following alteration in CNS homeostasis. This phenomenon is a physiologic mechanism to protect the CNS, resolve abnormalities, and restore homeostasis. Besides the neuroprotective and anti-inflammatory actions of monocyte-derived MФs and resident MФs, they can promote neuroinflammation by secretion of inflammatory cytokines and reactivation of primed T cells (37). In EAE, activation of microglia/MФs leads to disease progression (38). Also, in the CNS of MS patients, elevated myeloid cells and activated MФs have been found and associated with demyelination and axonal loss (39, 40). According to the role of MФs in neuroinflammation, they have attracted attention as a therapeutic target. Also, due to their dissimilar origin, location, and turnover, different strategies may require to target the various myeloid cell populations. As shown in multiple studies, direct targeting of myeloid cells has been shown to be effective in some other inflammatory diseases such as psoriasis, Crohn’s disease, and ulcerative colitis by targeting IL-12 and/or IL-23 (41, 42). Although MФs and their function in neuroinflammation have been described in detail in previous studies, their direct/indirect targeting by therapeutic agents has been less discussed. Here, we review the role of distinct subsets of MФs in the pathogenesis of MS and the impact of different therapeutic agents on these cells.

## The Role of Microglial Cells in Multiple Sclerosis Pathogenesis

Microglia are known as one type of glial cells and mononuclear phagocytes. These tissue resident cells are located in the brain and spinal cord. The number and location of these cells vary in different species, and human microglia dominate in white matter compared to gray matter (43). Microglia are developed from erythromyeloid progenitors (EMPs) in the yolk sac during primitive hematopoiesis (44), and their differentiation is regulated by some transcription factors such as IRF8, PU-1, and Runx-1 (45). Colony-stimulating factor 1 receptor (CSFR1) signaling is necessary for the survival of microglia, and its ligands, CSF1 and CD34, are produced in normal CNS (46).

Like MФs, these immune cells recognize infections, toxins, and injuries (47) and have a role in maintaining homeostasis in the adult CNS (48). Microglia use a specific signature called sensome in the homeostatic condition that scans changes in the CNS. So, they are the first cells that respond to damages in the CNS. Sensomes can recognize microorganisms and endogenous ligands. Some of the sensomes are specific integrins, purinergic receptors, and cluster differentiation (CD) markers, including P2ry12, Tumor Microenvironment of Metastasis 119 (TMEM119), Gpr34, CD33, CXCR4, and CX3CR1 (49). Studies in transgenic animals have shown that the interaction between CX3CR1 on microglia and MФs with fractalkine (CX3CL1) on neurons leads to the communication between immune and neural systems (50–53). Although microglia phenotype is considered resting or quiescent in stable and normal CNS, they have many functions (47, 54). Resting microglia influence surrounding cells through producing some neurotrophic factors such as insulin-like growth factor-1 (IGF-1), brain-derived neurotrophic factor (BDNF), TGF-β, and nerve growth factor (NGF) (55, 56). In addition, microglia participate in myelin debris removal and modulate neural activity and synaptic organization (57, 58). Moreover, they are involved in oligodendrocyte progenitor cell (OPC) maintenance in the CNS (59, 60) and partake in brain development through clearance of neuronal apoptotic bodies (61, 62). Advanced technologies such as single-cell RNA sequencing (scRNA-seq) and genetic fate mapping have improved the distinguishing of microglia subtype, function, and differentiation ways from MФs (63).

Jordão et al. (64) have used single-cell sequencing and found that in the homeostatic state, the microglia of EAE mice are distinguished into two subtypes, hMG1 and hMG2, and during inflammation, four populations [disease-associated microglia 1–4 (daMG)] have been observed. Furthermore, the gene profile of daMG demonstrates that they have more potential in chemokine production and subsequently disease progression compared to homeostatic parenchymal microglia (hMG) (64).

Microglia morphology in this situation is known as ramified. On the other hand, they have a long cytoplasmic protrusion for monitoring any changes in the CNS (47). This morphology is similar to the morphology of Langerhans cells in the skin (65). Due to their plasticity, microglia alter their phenotype under different conditions and environmental factors (66–68). They activate in response to the unstable state of the CNS (trauma, ischemia, or any threat in the CNS) and change their phenotype (69, 70). Like other innate immune cells, microglia recognize PAMPs and DAMPs through their pathogen recognition receptors (PRRs) (71–73). In this state, the morphology of activated microglia is known as amoeboid, which refers to cell mobility (74, 75). In addition, these cells are highly potent phagocytic cells that phagocytose dead cells and myelin debris (76).

Microglia, similar to MФs, show inflammatory and anti-inflammatory (alternatively) phenotypes in *in vitro* studies (77), and M2 phenotype microglia have subgroups including M2a, M2b, and M2c (78, 79). However, scRNA-seq and mass cytometry findings show that microglia phenotype and gene expression patterns are associated with age and regional differences (80).

According to previous findings, microglia’s role in MS pathogenesis is still unclear (76). Singh et al. (81) have shown that microglial nodules, which are the clusters of activated microglia, are present in the white matter of MS patients in the vicinity of plaques. They participate in response to axon degeneration and stressed oligodendrocytes (81, 82). Microglia and recruited MФs display a pro-inflammatory phenotype (M1 microglia) in the early MS and EAE disease stages. According to this phenotype, they have many functions, including oxidative injury, antigen-presenting, and T cell stimulating (76, 83). MФs and dendritic cells have more antigen presentation capacity to T cells than microglia in the early phases of EAE, but during inflammation, microglia express major histocompatibility complex (MHC)-II and costimulatory molecules that can stimulate T cells, so they act like an APC. Despite this ability, new MHC-II gene deletion experiments in microglia indicate that this population has no critical roles in EAE onset and progression (80, 84).

Furthermore, oxidative damage, which is mediated by reactive oxygen species (ROS), induces demyelination (85, 86). Many studies have indicated that innate immune-mediated oxidative injury (by activated microglia and other immune cells) has been proposed as an essential process underlying the progression of MS (87, 88).

Following scRNA-seq, Mendiola et al. (87) have demonstrated that in EAE, microglia are divided into five clusters according to the expression of genes involved in oxidative stress and Ag presentation. For example, cytochrome b-245 beta chain (Cybb), which encodes the nicotinamide adenine dinucleotide phosphate (NADPH) oxidase subunit, histocompatibility 2, class II antigen, and beta1 (H2-Ab1) gene that participate in MHC-II expression are microglial clusters during oxidative stress and Ag presentation. Also, the ability of different clusters of microglia varies in oxidative damage and antigen presenting. So, the MgV cluster is more involved in the oxidative injury, while the MgIII cluster is enriched for Ag presentation (87).

**Figure 1:**
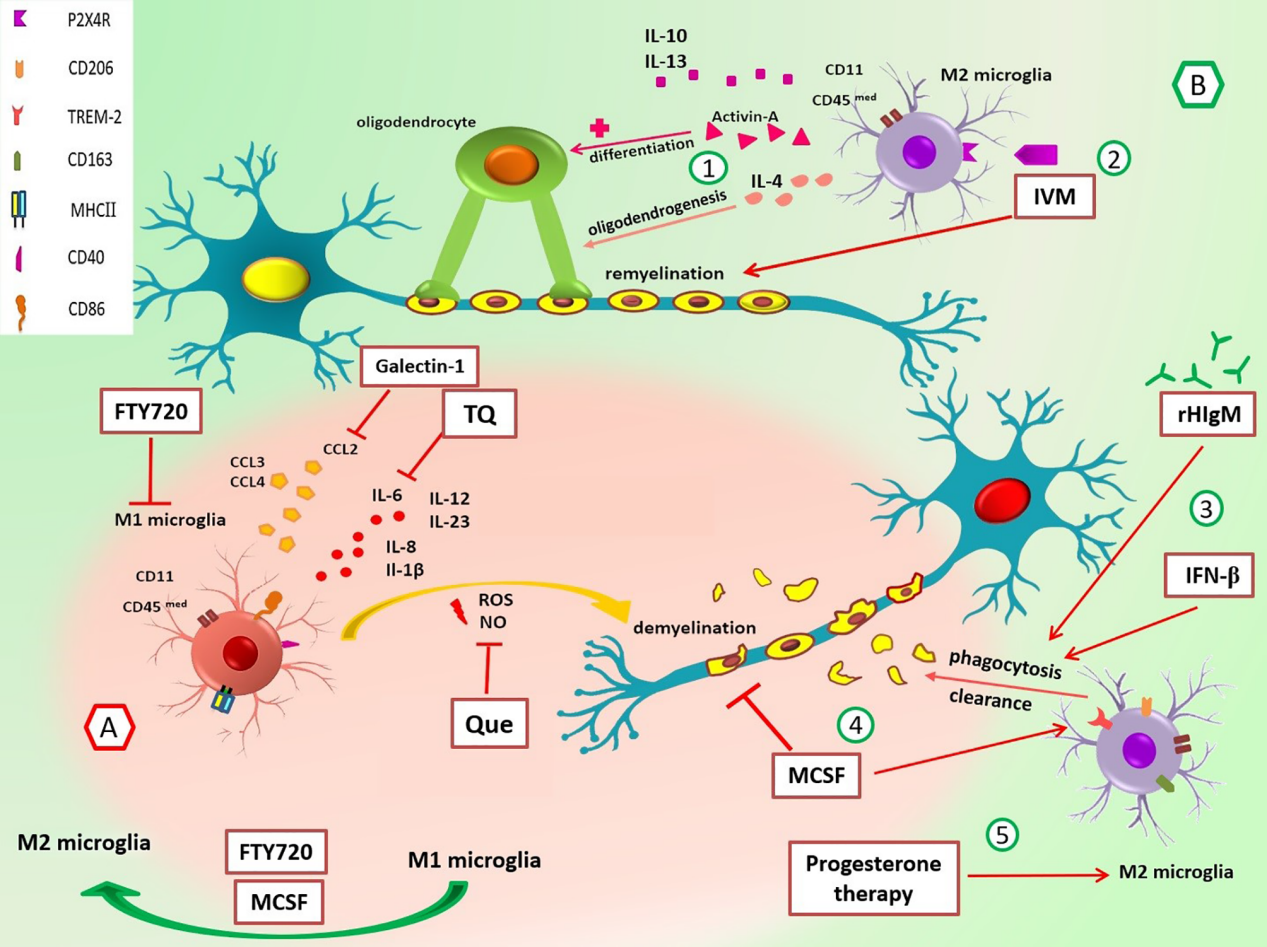
Roles of M1/M2 microglia and the effect of different drugs on these cells in multiple sclerosis. **(A)** In the early stage of EAE and MS, M1 MG have different roles in the promotion of inflammation through cytokine/chemokine release, and ROS and NO production leads to demyelination. Suppression of M1 MG and their functions can be useful in MS control. For example, galectin-1 decreases the production of CCL2, TQ reduces IL-6, Que decreases the release of NO, and FTY-720 suppresses MG activation and switches M1 to M2 phenotype. **(B)** M2 MG have anti-inflammatory functions and promote remyelination *via* cytokines release and phagocytosis of myelin debris. 1) IL-4 promotes oligodendrogenesis, and activin-A helps differentiation of oligodendrocytes. 2) IVM interacts with P2X4R and enhances phagocytosis and remyelination. 3) rHIgM22 and rIFN-β promote phagocytosis and myelin uptake. 4) M-CSF increases the expression of TREM2 mRNA, diminishes demyelination, and improves the organization of myelin sheaths through polarization of M1 MG to M2 MG. 5) Progesterone therapy increases marker expression of M2 phenotype (CD206). CCL, chemokine (C-C motif) ligand; CD40, cluster of differentiation 40; CD86, cluster of differentiation 86; CD163, cluster of differentiation 163; CD206, cluster of differentiation 206; EAE, experimental autoimmune encephalomyelitis; FTY720, fingolimod; IL, interleukin; IVM, ivermectin; M-CSF, macrophage colony-stimulating factor; MG, microglia; MHC-II, major histocompatibility complex class II; NO, nitric oxide; P2X4R, P2X4 receptor; Que, quetiapine; rHIgM22, recombinant human IgM; rIFN-β, recombinant interferon-beta; ROS, reactive oxygen species; TQ, thymoquinone; TREM-2, triggering receptors on myeloid cells-2.

Activation of microglia also induces the expression of different transcription factors such as nuclear factor (NF)-κB, Janus kinase (JAK)/STAT, c-Jun N-terminal kinase (JNK), extracellular signal-regulated kinase (ERK)1/2, and p38. Moreover, different cytokines, including IL-6, IL-8, IL-12, IL-23, IL-1β, and TNF, are produced after microglia activation (89, 90). In addition, the induction of chemokines such as CCL2, CCL3, and CCL4 also is induced by activated microglia, which can facilitate leukocyte recruitment in the early phase of EAE (91) (**Figure 1**).

Oxidative processes and pro-inflammatory cytokines result in injury to oligodendrocytes (76). Heppner et al. (92) have shown that microglia paralysis of transgenic mice ameliorates inflammation in the CNS and improves clinical symptoms of the disease. Also, Bhasin et al. (93) have demonstrated that microglia inhibition at the onset of EAE attenuates disease signs and decreases EAE progression.

Autophagy is a conserved homeostatic pathway in eukaryotic cells, which has recently become evident in neurodegenerative disorders (94). There is a consideration that autophagy is associated with the regulation of inflammation in microglia during neuroinflammation (95). Many studies revealed that following autophagy induction in inflammatory microglia, the expression of inflammatory genes is suppressed and anti-inflammatory phenotype is promoted (96–98). In EAE mice, induction of autophagy leads to inflammasome inhibition and attenuation of symptoms (99). Also, Atg5 knockdown in microglia leads to more neuroinflammation in cell culture (98, 100). Moreover, ATG is involved in remyelination and debris cleaning in microglia (101, 102).

The ratio of M1/M2 is an essential factor in the relapse of EAE, and M1 microglia is more than M2 in the early phase of repair. Environmental changes can shift phenotype; however, underlying mechanisms responsible for this switch are unknown (83, 103, 104). M2 microglia play an essential role in the recruitment and differentiation of oligodendrocyte progenitor cells (OPCs) through the clearance of myelin debris. An *in vitro* study has shown that M2 cell medium inhibits OPC apoptosis even in the absence of serum and growth factors. Also, evaluation of myelin basic protein (MBP) and myelin oligodendrocyte glycoprotein (MOG) reveals that M2 microglia promote oligodendrocyte differentiation (103, 105). M2-produced anti-inflammatory cytokines (IL-4, IL-10, and IL-13) and substances such as activin-A are involved in differentiation of oligodendrocyte during remyelination (103, 104, 106, 107) (**Figure 1**). Also, Miron et al. (103) have indicated that blocking antibodies against M2 cell-derived activin-A diminishes oligodendrocyte differentiation.

Furthermore, anti-inflammatory cytokines such as IL-4 promote oligodendrogenesis; thus, it is helpful for remyelination (108). In contrast, the protective function of TNF as a pro-inflammatory cytokine has been shown in EAE (109). Accordingly, transmembrane TNF (tmTNF) and TNFR2 induce remyelination in EAE, while soluble TNF (solTNF) suppressed phagocytosis of myelin debris and thus inhibited remyelination in the cuprizone demyelination model (110).

MicroRNAs (miRNAs) are a group of small non-protein-coding RNAs, which have a role in biological functions through the regulation of gene expression. Different miRNAs can affect microglia and MФ functions. Mir-124 is a specific miRNA in the brain and plays a role in CNS development and neurogenesis of adults (111, 112). Mir-124 is highly expressed in microglia compared to other cells and can maintain the resting phenotype of microglia. In experimental studies, no evidence of microglial activation has been shown in EAE mice treated with mir-124. Ponomarev et al. (112) have found that transfection of bone marrow-derived macrophages (BMDMs) with mir-124 induced downregulation of markers such as CD45 and CD11b, suppressed the expression of TNF-α and iNOS, and increased the expression of anti-inflammatory cytokine TGF-β. Moreover, they have indicated that inflammatory responses and EAE symptoms were alleviated in treated mice (112).

Long intergenic noncoding RNA (lincRNA)-Cox2 belongs to long noncoding RNA and can regulate immune functions. LincRNA-Cox2 plays a role in inflammatory responses through binding to the p65 subunit of NF-κB and modulating NLRp3 and Asc expression. Xue et al. (113) showed that knockdown of lincRNA-Cox2 promoted resting microglia (CD11b+ CD45med) and suppressed IL-1β secretion. Also, lincRNA-Cox2 silencing inhibited NLRP3 inflammasome activation and thereby promoted autophagy in BMDMs and microglia. Moreover, knockdown of lincRNA-Cox2 in EAE models decreased inflammatory cells in the white matter and improved EAE symptoms.

Collectively, evidence indicates that activated microglia act as a double-edged sword in MS pathogenesis (38). So, targeting microglia activation and inducing a shift to M2 phenotype would be a promising choice in the future of MS treatment.

## Microglia and Macrophages Markers

Resting microglia do not highly express MHC-II and costimulatory molecules, so they cannot prime T cells (114, 115). The expression of surface markers changes following activation of microglia. For example, myeloid marker expression and adenosine A2A receptors are upregulated during their activation, while P2Y12 receptors are downregulated (54, 116, 117). Also, MHC expression and costimulatory molecules such as CD80, CD86, and CD40 have been increased after microglia activation in EAE (106, 118, 119). However, specific deletion of MHC-II in microglia does not promote disease progression, so microglia is not enough to stimulate autoreactive T cells (120). Moreover, studies have shown that microglia are impaired APCs despite their ability to uptake myelin (121, 122).

In the inflammatory state, microglia express p22phox, CD68, CD86, and MHC-II antigens, while in the inactive lesion, they mostly express anti-inflammatory markers including CD206, CD163, and ferritin (76, 123). Efficient myelin debris removal and clearance by phagocytosis is an essential step in effective remyelination, and the surface expression of triggering receptors on myeloid cells-2 (TREM2) plays a key role in phagocytosis (124). Piccio et al. (125) have indicated that the expression of TREM2 on microglia is increased during EAE, and blocking of this receptor with mouse monoclonal antibody is accompanied by cellular infiltration and EAE exacerbation. Also, other molecules such as complement receptor 3 (CR3), signal regulatory protein (SIRP), IFN-β, and transmembrane TNF (tmTNF) participate in this process (106, 110, 126). Discriminating microglia from MФs is challenging; however, some markers such as CD45 and CD11b have been introduced as differential markers.

According to this classification, CD11b+ CD45med cells are microglia, and CD11b+CD45hi cells are MФs (127); however, this classification is controversial, and the expression of some markers such as CD45 changes under different conditions (127–130). Furthermore, there are more reliable differential markers, including TMEM119, Sal-like1 (Sall1), sialic acid-binding Ig-type lectin H (Siglec-H), and P2Y12R (76, 131–136).

TMEM119 is a cell-surface protein that is highly expressed on human and mouse microglia. This protein indicates a highly conserved sequence and does not express on MФs and immature microglia; however, its function is still unknown (131). The purinergic receptor (P2Y12) directs microglia movement toward damage sites (137). The other molecule, Sall1, which is a transcriptional regulator, plays a role in microglia morphology and gene expression (134). Siglec-H is mainly expressed on microglia in mice, but the homology of human Siglec-L2 with Siglec-H is approximately 40% (138, 139).

## Monocyte-Derived Macrophages in the Central Nervous System

Peripheral blood monocytes are derived from bone marrow hematopoietic stem cells (HSCs) and defined as classical (CD14+CD16-), non-classical (CD14lowCD16+), and intermediate monocytes. However, there are few infiltrating monocytes in the CNS under physiological conditions. Also, substantial accumulation of monocytes, predominantly non-classic CD16+, in both gray and white matter MS lesions is significant, especially during disease relapses (140, 141). During the effector stage of EAE, monocytes rapidly infiltrate surrounding meninges, perivascular space, and choroid plexus through and differentiate into MФs (142, 143). These MФs contribute to the progression of the paralytic stage of EAE and demyelination by expressing MHC-II, costimulatory molecules, and producing pro-inflammatory factors (38). Thus, in EAE, MФ depletion is associated with a lower CNS injury and attenuated signs and symptoms of disease (144, 145).

Expression of cell adhesion molecules such as intercellular adhesion molecule (ICAM)-1, vascular cell adhesion molecule (VCAM)-1, and activated leukocyte cell adhesion molecule (ALCAM) by CNS endothelial cells and their interaction with integrins like leukocyte function-associated antigen [(LFA)-1, αLβ2], VLA-4 (α4β1), and CD6 are essential steps of immune cell migration into the CNS (146). Nerve injury-induced protein (Ninjurin)-1 and junctional adhesion molecule-like (JAML) are other adhesion molecules involved in monocyte-derived MФ migration (147, 148).

Moreover, CCR2 is a crucial chemokine receptor in the recruitment of Ly6Chigh monocytes to the inflamed CNS, which exacerbates disease progression in the EAE model. So that mice without CCR2 are resistant to EAE induction (149). Besides, CCR4, a chemokine receptor for CCL17 and CCL22, is upregulated in MФs of CNS lesions, and interestingly, mice lacking CCR4 have also been reported to be resistant to EAE (150).

Both M1 and M2 MФs are detected in MS lesions, and they may repolarize to apposite phenotype depending on the local environment and stage of disease. According to The study by Vogel et al. in active and chronic active MS lesions, the expression of typical M1 markers is higher than M2 markers (151). Also, in EAE, both M1 and M2 MФs enhance and regulate the disease’s pathogenesis (152, 153).

**Figure 2:**
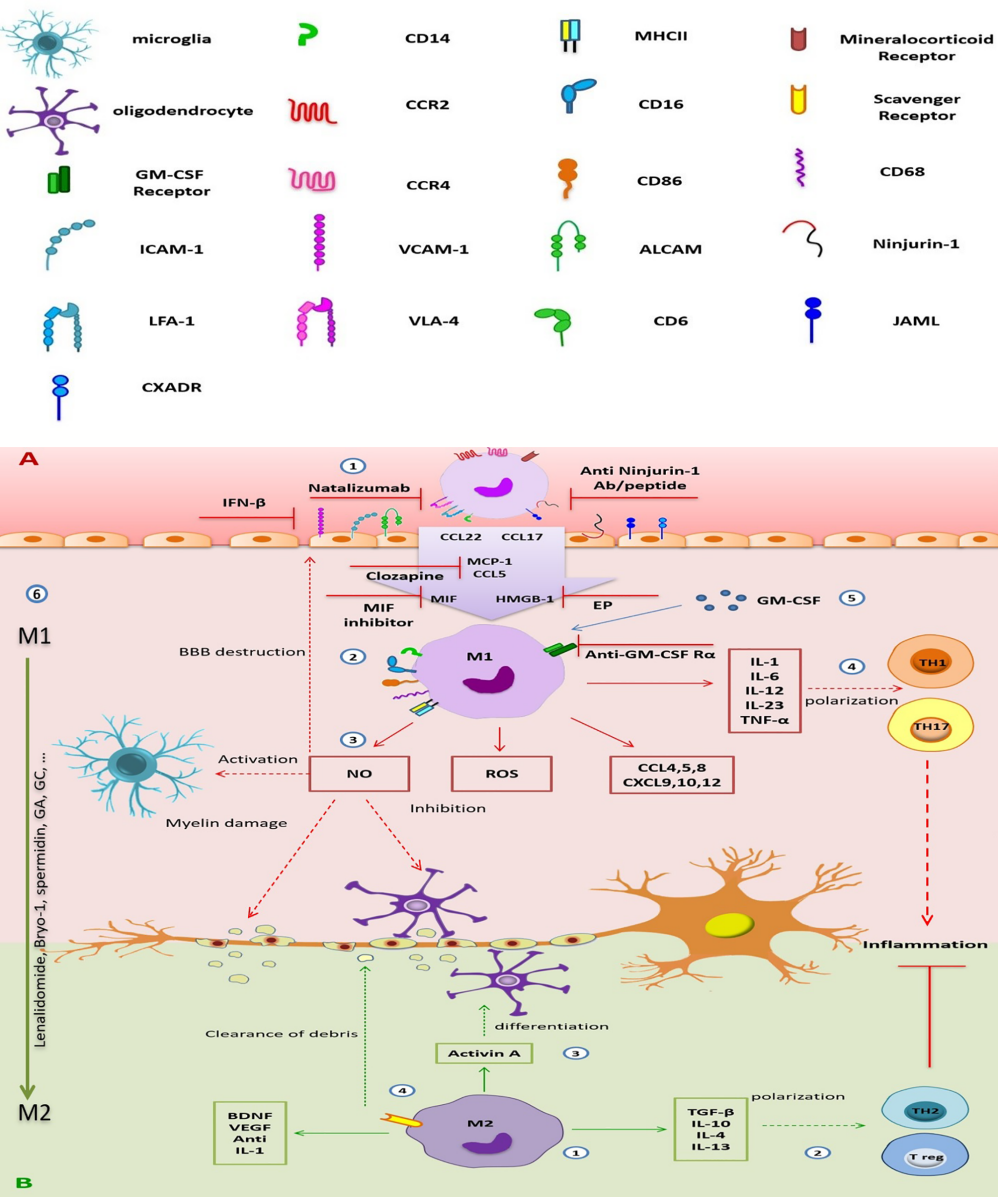
The destructive/regenerative roles of M1/M2 macrophages in multiple sclerosis and possible treatments. **(A)** 1) Peripheral blood monocytes enter the inflamed CNS following the attachment to adhesion molecules (e.g., the interaction of ICAM with LFA-1, VCAM-1 with VLA-4, ALCAM with CD6, homophilic interaction of ninjurin-1 and JAML with themselves, and also JAML with the other receptor CXADR), the concentration gradient of chemokines [CCL5, CCL17, CCL22, and MCP-1(CCL2)], MIF, and HMGB-1 through damaged BBB, and differentiated into monocyte-d MФs. Inhibition of adhesion molecules (e.g., ICAM-1 and VCAM-1 by IFN-β, VLA-4 by natalizumab, and ninjurin-1 by anti-ninjurin-1 blockade), receptors (e.g., MR), or chemokines and other stimulators (e.g., MCP-1 by clozapine or HMGB-1 by EP), which is involved in monocyte migration could be a therapeutic approach. 2) M1 MФs (CD86+, CD68+, MHC-II+) are the dominant subpopulation of monocyte-d MФs. They enhance CNS inflammation by producing pro-inflammatory cytokines, chemokines, ROS, and NO. 3) NO production leads to increase BBB destruction, microglial activation, myelin damage, and inhibits oligodendrocyte function. 4) Pro-inflammatory cytokines are involved in TH1 and TH17 polarization, enhancing neuroinflammation. 5) GM-SCF is essential for differentiation and function of M1 MФs, so, GM-CSFR blockade can improve inflammation. 6) Repolarization of inflammatory M1 MФs into anti-inflammatory M2 phenotype could be a good choice for MS treatment. **(B)** 1) A smaller population of monocyte-d MФs is M2 MФ with anti-inflammatory phenotype. It secretes immunomodulatory cytokines, chemokines, and tissue regenerative agents. 2) Anti-inflammatory cytokines induce the polarization of TH2 and Treg cells, which suppress neuroinflammation. 3) Secreted activin A leads to oligodendrocyte differentiation. 4) Expression of scavenger receptor is involved in cleaning the myelin debris. CNS, central nervous system; ICAM-1, intercellular adhesion molecule-1; VCAM-1, vascular cell adhesion molecule-1; ALCAM, activated leukocyte cell adhesion molecule; LFA-1, leukocyte function-associated antigen-1; VLA-4, very late antigen-4; CD6, cluster of differentiation 6; ninjurin-1, nerve injury-induced protein-1; JAML, junctional adhesion molecule-like; CCL2, chemokine (C-C motif) ligand 2; CCL17, chemokine (C-C motif) ligand 17; CCL22, chemokine (C-C motif) ligand 22; CCL3, chemokine (C-C motif) ligand 3; CCL4, chemokine (C-C motif) ligand 4; CCL5, chemokine (C-C motif) ligand 5; CCL8, chemokine (C-C motif) ligand 8; CXCL9, chemokine (C-X-C motif) ligand 9; CXCL10, chemokine (C-X-C motif) ligand 10; CXCL12, chemokine (C-X-C motif) ligand 12; MCP-1, monocyte chemoattractant protein-1; MIF, macrophage migration inhibitory factor; HMGB-1, high-mobility group box-1; BBB, blood–brain barrier; MФ, macrophage; MR, mineralocorticoid receptor; CD86, cluster of differentiation 86; CD68, cluster of differentiation 68; MHC-II, major histocompatibility complex class II; ROS, reactive oxygen species; NO, nitric oxide; TH1, T helper type 1; TH17, T helper type 17; TH2, T helper type 2; Treg, regulatory T cell; IL-1, interleukin-1; IL-6, interleukin-6; IL-12, interleukin-12; IL-23, interleukin-23; IL-10, interleukin-10; IL-4, interleukin-4; IL-13, interleukin-13; TNF-α, tumor necrosis factor-alpha; TGF-β, transforming growth factor-beta; GA, glatiramer acetate; EP, ethyl pyruvate; GM-CSFR, granulocyte-macrophage colony-stimulating factor receptor; VEGF, vascular endothelial growth factor.

During MS, M1 MФs secrete high amounts of pro-inflammatory agents such as IL-6, IL-12, IL-1, TNF-α, IL-23, reactive oxygen species, and nitrogen species and CCL4, CCL5, CCL8, CXCL9, CXCL10, and CXCL2. This condition leads to the recruitment of immune cells, exacerbating neuroinflammation and tissue damage (142). IL-6 is a crucial cytokine in CNS autoimmunity establishment, as IL-6-deficient mice have shown attenuated EAE symptoms. Furthermore, IL-1β has been considered as an inducer of Th17 polarization and EAE progression (154). Recent research on bone marrow chimeric mice has revealed that monocyte-derived MФs express TRPM2 protein and subsequently produce CXCL2, leading to enhanced neutrophil infiltration and EAE progression (155). Studies on brain autopsy of MS patients have shown that M1 MФs express CD68 (as a phagocytosis marker), HLA, and CD86, which contribute to antigen-presenting to primed T cells. Also, iNOS has increased in M1 MФs. iNOS enzyme and nitric oxide production have an important impact on microglia activation, BBB disruption, demyelination, oligodendrocyte injury, axonal degeneration, and axonal conduction impairment (76, 156). According to single-cell oxidative stress transcriptome analysis of CNS innate immunity in EAE, similar to microglia, seven monocyte/MФ clusters (MpI–VII) have been identified, which have different potentials in ROS production and Ag presentation. Regarding the results, Clusters MpI and MpII had increased Cybb and H2-Ab1 expression, whereas clusters MpIII and MpIV had only high expression of H2-Ab1 and are more potent in Ag presentation (87). So, according to previous studies, the M1 MФs are generally considered harmful in MS (**Figure 2**).

On the other hand, studies have demonstrated the neuron-protective activities of MФs in EAE. High levels of M1 MФ infiltration present in the CNS during exacerbations of disease in mice, but a gradual increase in M2 MФs is associated with improved neurological impairment (157). The increase in the expression of tissue transglutaminase (TG2) mRNA level in monocytes derived from MS patients indicates anti-inflammatory MФs and subsequently immunomodulatory cytokines (158). M2 MФs cause an anti-inflammatory state and tissue repair by secreting IL-4, IL-10, IL-13, and TGF-β cytokines. These cells also drive the recruitment and differentiation of Th2 and regulatory T cells (Treg), which suppress the inflammatory response in EAE mice (159).

Moreover, M2 MФs express scavenger receptors to clear myelin debris in the damaged spinal cord, promoting CNS repair (160). These populations of MФs can produce neurotrophic factors, including IGF-1, BDNF, vascular endothelial growth factor (VEGF), epidermal growth factor (EGF), and IL-1 receptor antagonist that leads to alleviate sympathetic neuron dysfunction (161). Also, they block the iNOS enzyme to decrease inflammation, increase environment stability, and protect neural cells against injury (162) (**Figure 2**). In summary, M2 MФs dominantly play a role in suppressing inflammation and promoting tissue regeneration. However, the dichotomy of MФ polarization is not accurate. Accordingly, in the active MS lesion, the presence of MФs with an intermediate phenotype, co-expressed M1 and M2 markers, has been confirmed. So, it seems that MФ phenotype and function are influenced by environmental conditions (151). In the following, we will discuss the effects of different therapeutic agents on MФs and microglia in the CNS of MS patients.

### Actual Therapeutic Approaches That Affect Macrophages and Microglia Population in Multiple Sclerosis

DMTs are a group of drugs that reduce the early clinical and subclinical disease activity that may contribute to long-term disability. More than 10 Food and Drug Administration (FDA)-approved DMTs target the immune-mediated disease process and differ in routes of administration in addition to their frequencies (163). Generally, T cells and B cells are most frequently discussed as targets of DMTs, but some of the current MS disease-modifying therapies also affect myeloid cells, although these cells are not the main target of the drug (164). The probable effects of DMTs on microglia and monocyte-derived MФs have been shown in **Table 1**, and some of them are discussed below:

**Table 1:** Probable effects of disease-modifying therapies (DMTs) on microglia and/or monocyte-derived macrophages.

DMTs	Definition	Effect on microglia and/or monocyte-derived macrophages
**Interferon-β**	Cytokine released by host cell in response to viral infection and regulating immune responses (165)	- In MS patients, induces anti-inflammatory phenotype by reducing the production of nitric oxide and in contrast, increasing the expression of BDNF and Ig like transcript-3 in monocyte-derived MФs (166–168). Inhibits infiltration of monocyte-derived MФs into the CNS (169). - In EAE, upregulating IL-27 expression in monocyte-derived MФs leads to Th17 suppression (170) - *In vitro*, promotes phagocytosis capacity of microglia (126).
**Glatiramer acetate**	Synthetic amino acid polymer (15, 171)	- In MS, induces anti-inflammatory phenotype by inhibiting the production of nitric oxide in both microglia and monocyte-derived MФs. Enhances the phagocytic activity of microglia and monocyte-derived MФs (172, 173). Decreases microglial activation (174). - In EAE, promotes anti-inflammatory phenotype in monocyte-derived MФs by increasing the production of IL-10 and TGF-β and decreasing the secretion of pro-inflammatory cytokines and the expression of adhesion molecules (175). - *In vitro*, increases the production of IL-10 and reduces TNF-α in microglia (176).
**Fingolimod**	Agonist of sphingosine-1-phosphate (S1P) receptor (177)	- In MS, induces anti-inflammatory phenotype by inhibiting the production of pro-inflammatory cytokines and expression of pro-inflammatory miR-155 in monocyte-derived MФs. (116/1, 164.167/2) - In EAE, decreases CD40 expression and production of TNF in monocyte-derived MФs (178). - *In vitro*, switches M1 microglia to M2 phenotype (177).
**Natalizumab**	Anti-VLA-4 humanized monoclonal antibody (18)	- In MS, reduces microglia activation (179). - In EAE, suppresses the activated microglia and monocyte-derived MФs (180).
**Dimethyl** **Fumarate**	Methyl ester of fumaric acid (181)	- In MS, decreases the expression of pro-inflammatory mir-155 in monocyte-derived MФs (182). - In EAE, reduces the infiltration of monocyte-derived MФs in to the CNS (183) - *In vitro*, induces anti-inflammatory phenotype by inhibiting the production of nitric oxide and pro-inflammatory cytokines in microglia (184).
**Teriflunomide**	A reversible inhibitor of mitochondrial enzyme dihydrooratate dehydrogenase (DHODH) (185)	- In MS, induces anti-inflammatory phenotype by increasing the production of IL-10 and PDL-1 expression (186). - In EAE, inhibits the migration of monocyte-derived MФs in to the CNS (187, 188). - *In vitro*, induces anti-inflammatory phenotype in microglia by increasing IL-10 production (187, 189).
**Rituximab**	Chimeric Anti-CD20 monoclonal Ab (190)	- Inhibits monocyte activation by depleting GM-CSF expressing memory B cells (190).
**Mitoxantrone**	Cytotoxic agent of the anthracenedion family (191)	- *In vitro*, reduces migration capacity of monocytes (192).
**Siponimod**	Selective sphingosine-1-phosphate receptor modulator (193)	- In EAE, reduces the production of IL-6 and CCL5 in activated microglia (194) - *In vitro*, inhibits IL-6 production in siponimod-treated microglia (193).
**Cladribine**	Chlorodeoxyadenosine (CdA), is purine nucleoside analog (195)	- *In vitro*, inhibits the proliferation of microglia. Induces apoptosis in microglia (195)

MS, Multiple sclerosis, BDNF, Brain-derived neurotrophic factor; Ig, Immunoglobulin; CNS, Central nervous system; EAE, Experimental autoimmune encephalomyelitis; TH17, T helper type 17; IL-27, Interleukin-27; IL-10, Interleukin-10; TGF-β, Transforming growth factor-beta; TNF-α, Tumor necrosis factor-alpha; miR-155, microRNA-155; CD-40, Cluster of differentiation 40; VLA-4, Very late antigen-4; PD-L1, Programmed death-ligand 1; CD-20, Cluster of differentiation20; Ab, Antibody; GM-CSF, Granulocyte-macrophage colony-stimulating factor; IL-6, Inreleukin-6; CCL5, chemokine (C-C motif) ligand 5.

IFN-β is a member of the human type I interferons family that has different roles in the regulation of the immune system, including the decrease of tissue damage and inflammation through downregulation of matrix metalloproteinase 9 (MMP-9), inhibition of effector cell migration by downregulating the adhesion molecule VLA-4, and prevention of T-cell proliferation (196–198). Besides, IFN-β decreases cell migration to the CNS through CCR7 inhibition and reduces pro-inflammatory cytokines such as IL-12 in monocytes (199, 200).

This cytokine is the first FDA-approved drug used in the treatment of RRMS to reduce relapses and severity of MS disease due to its various immunomodulatory properties and several actions on immune cells (201, 202).

Kocur et al. (126) have found that IFN-β-treated microglia accumulate in areas containing myelin debris for phagocytosis. Moreover, adult wild-type and IFN-β−/− mice microglia and BV2 microglia in culture media promote phagocytosis of myelin debris after treatment with recombinant IFN-β (rIFN-β), while IFNAR1−/− microglia show a bit of a promotion. Therefore, IFN-β and IFNAR1signaling are necessary to stimulate microglial phagocytosis of myelin debris (126) (**Figure 1**). Another study by Floris et al. (169) in IFN-β-treated EAE animals has shown reduced clinical score and improved disease symptoms. Furthermore, they have found that following this treatment, expressions of ICAM-1 and VCAM-1 were reduced in the CNS endothelial cells, leading to the subsequent reduction in monocyte-derived MФ migration into the inflamed CNS (169) (**Figure 2**).

The other therapeutic agent, glatiramer acetate (GA, Copolymer-1, Copaxone), is a drug that affects MФs. It is prescribed in RRMS, and its clinical effects have been indicated in both MS and MS models (203). Weber et al. have addressed one of the immunological mechanisms of GA treatment in EAE mice. They have found that GA can develop anti-inflammatory type II monocyte polarization with an increase in the production of IL-10 and TGF-β. It also decreases the secretion of IL-12 and TNF-α and the expression of CD40 and CD80. Furthermore, GA-treated type II monocytes can reverse clinical EAE, accompanied by a reduction in the number of CNS lesions. This GA mechanism has shown the importance of type II monocytes in the future of drug intervention in MS (175) (**Figure 2**).

Fingolimod (FTY720) is an FDA-approved drug for the treatment of RRMS. It is a high-affinity agonist of sphingosine-1-phosphate (S1P) receptor, with an immunosuppressive effect. Qin et al. (177) have reported that fingolimod (FTY720) suppresses microglial activation (fewer Iba-1+ or CD68+ microglia) and attenuates neuroinflammation in a mouse model of white matter (WM) ischemic damage caused by chronic hypoperfusion. It switches microglial polarization from M1 to M2 phenotype in WM ischemic injury through activating STAT3 (177). Furthermore, other studies have shown that fingolimod influences MФs, and monocytes induce switching to M2 phenotype in culture and decrease IL-12 production (199) (**Figure 1**).

Another DMT is natalizumab, a humanized monoclonal antibody used in the treatment of RRMS and reduces relapse rate and axonal damage. This Ab binds to α4 subunit of α4β7 integrin, and actually, it can inhibit adhesion molecule VLA-4, which has a role in the pathogenesis of EAE and MS (18). Mindur et al. (180) have shown that natalizumab can suppress the activated microglia and MФs in the onset of EAE. Also, studies have demonstrated that monocyte-derived MФs entered into the CNS using VLA-4 so that anti-VLA-4 may decrease MФ infiltration to the CNS (180) (**Figure 2**). Moreover, Sucksdorff et al. (179) have reported that natalizumab can decrease microglia activation in normal-appearing white matter and at chronic active lesions of MS patients’ brains. In another study, Öhrfelt et al. (204) indicated that the CSF-soluble TREM2, a marker of microglial activation, is reduced to baseline levels in MS patients following treatment with natalizumab. But the exact effect of this Ab on microglia is not understood (204).

### Promising Therapeutic Approaches That Affect Macrophages and Microglia Population in Multiple Sclerosis

#### Inhibition of Migration and Infiltration of Immune Cells to the Central Nervous System

Nerve injury-induced protein-1 (ninjurin-1) is a cell surface protein that is found in many tissues such as CNS vascular endothelial cells and leukocytes (remarkably in monocytes), leading to an interaction between these cells in a homophilic manner (205). As Ifergan et al. showed, the expression of ninjurin-1 was upregulated in inflammatory APCs in the CNS of EAE mice and in MS lesions. So, it is associated with the migration of monocytes across the brain endothelium. Furthermore, this group found that blockade of ninjurin-1 with either the Ab or the peptide resulted in alleviating EAE symptoms and reducing demyelination and immune cell infiltration in mice (147). According to this result, ninjurin-1 targeting may be helpful in MS treatment (**Figure 2**).

The chemokines, including monocyte chemoattractant protein 1 (MCP-1 or CCL2) and CCL5 or RANTES (Regulated on Activation, Normal T cell Expressed and Secreted), are expressed by different cell types in the CNS and secreted by infiltrating blood-derived MФs following their infiltration into the CNS. These chemokines are associated with acute symptoms of CNS disease in rats and mice (206, 207). Recently, Robichon et al. (208) have treated EAE mice with clozapine, an atypical antipsychotic agent and can cross the BBB (209). They have indicated that clozapine reduces the infiltration of monocytes, neutrophils, and T cells by decreasing the expression of CCL2 and CCL5 in the CNS. This agent also directly upregulates cyclic AMP in immune cells, which leads to alteration of CCL5 and CCL2-mediated signaling pathways and inhibition of migration. As CCL2 and CCL5 are involved in MФ migration and regulation in EAE, drugs such as clozapine that target CCL2 and CCL5 expression should be considered in future studies (208) (**Figure 2**).

Ethyl pyruvate (EP) is the other compound, a redox analog of dimethyl fumarate (Tecfidera). In a study, Djedović et al. (210) have shown that EP decreases the EAE symptoms at the time of disease peak by inhibiting high-mobility group box 1 protein (HMGB1) in ED1+ and Iba1+ reactive microglia. This effect is induced by reducing the degeneration of axons (210, 211) (**Figure 2**).

Also, mineralocorticoid receptor (MR or NR3C2) has immunoregulatory effects and plays an important role in developing the polarization of myeloid cells toward the inflammatory M1 phenotype (212). Montes-Cobos et al. (213) have deleted the expression of this receptor in myeloid cells in EAE mice (MrlysM Mice) and showed that it is accompanied by reducing neuroinflammation and frequency of inflammatory monocytes and microglia (CD45high CD11bhigh Ly6Chigh) in the CNS. Also, the onset of the disease in MrlysM Mice and control populations was similar, but in the mutant mice, in the chronic phase of the disease, the severity has been significantly reduced. Based on these results, blockade of MR by different drugs has the potential improvement effects in MS disease (213) (**Figure 2**).

Macrophage migration inhibitory factor (MIF) is a pro-inflammatory cytokine that is associated with various inflammatory diseases. Its elevation has been identified in the CSF of patients during a relapse of MS (214). Kithcart et al. have observed that administering an MIF inhibitor to C57Bl/6 mice protects them from EAE. Furthermore, they have found little or no infiltration of MФs in the spinal cord. They also have found that MIF-deficient C57Bl/6 mice have significantly fewer severe clinical signs of disease during both the acute and chronic phases of the disease. Therefore, MIF inhibitors or MIF deletion could be a novel therapeutic option for MS treatment (215) (**Figure 2**).

#### Targeting the Activation and Function of Microglia and Macrophages

Galectin-1 is a family of endogenous lectins encoded by the Lgals1 gene. Starossom et al. (216) have found that recombinant galectin-1 decreases surface expression of MHC-II, CD86, and iNOS mRNA in microglial cells *in vitro*. Galectin-1 also diminishes the production of TNF and CCL2 levels in IFN-γ-polarized M1 microglial cells. Moreover, induction of EAE in Gal1-deficient (Lgals1-/-) mice has led to an increase in Iba+ MHC-II+ microglial cells and axonal loss and a decrease in axonal outgrowth during autoimmune neuroinflammation. Interestingly, the adoptive transfer of Gal1-secreting astrocytes to these mice has suppressed EAE by inhibiting microglia (216). So, galectin-1 is a critical molecule in the regulation of microglia and can be considered in treating neuroinflammation diseases (**Figure 1**).

Quetiapine (Que) is an atypical antipsychotic drug (APD), and previous studies have indicated that APDs influence activated microglia through the reduction of TNF-α and nitric oxide (NO) production (217–219). Que regulates immune responses in EAE by suppressing the release of pro-inflammatory factors from activated microglia (218, 220).

Wang et al. (221) have used long-term Cuprizone-treated mice (mimics the chronic phase of neuroinflammation disease) and treated them with Que. They have found Que inhibits the activation of microglia/MФs in corpus callosum lesions. Also, pretreatment with Que inhibits the translocation of NF-κB p65 subunits and Ca2+ elevation by reducing the upregulation of STIM1 and modulation of store-operated Ca2+ entry (SOCE). Since the Ca2+ signaling pathway is significant for microglial activation (221), Que probably influences these cells through the above mechanisms. Thus, Que and other drugs that affect calcium channels and regulate microglial activity could be incorporated into new research (**Figure 1**).

Moreover, thymoquinone (TQ), which is extracted from the *Nigella sativa* plant seed oil, has reduced inflammatory cytokines such as IL-2, IL-4, IL-6, IL-10, and IL-17a in LPS/IFN-γ-activated microglia. In addition, it has downregulated several NF-κB signaling target genes, including IL6, complement factor B (CFB), CXCL3, and CCL5. Furthermore, TQ treatment has increased neuroprotective protein expression in LPS/IFN-γ-activated BV-2 microglial cells (222–225) (**Figure 1**).

Like microglia, some therapeutic agents influence the functional activity of monocyte-derived MФs. Accordingly, GM-CSF is a cytokine that plays a critical role in neuroinflammation onset, as GM-CSF KOs are resistant to disease induction (226). Ifergan et al. (227) have found that targeting the GM-CSF receptor (expressed on monocytes, DCs, MФs, and neutrophils) can alleviate chronic EAE. Its blockade has resulted in a significant reduction of the relapse severity in treated mice compared to controls (227). Furthermore, following anti-GM-CSF Rα treatment, the costimulatory molecules such as CD80, CD86, CD40, and MHC II expression and inflammatory cytokines, including IL-1β, IL-6, IL-12p40, IL-23p19, and TNF-α by mDCs and inflammatory monocytes, have reduced. Also, in the presence of anti-GM-CSF Rα, chemotactic agents are required in inflammatory monocyte migration like CXCR2 (binds to MIF) and CCR6 decrease and ameliorate EAE. Moreover, Lotfi et al. (228) have indicated that GM-CSF blockade in monocytes is accompanied by CXCL11 production and T-cell suppression *in vitro*. Because CNS-infiltrating inflammatory monocytes and mDCs highly express GM-CSF Rα in both EAE and MS, anti-GM-CSF Rα treatment could be a good suggestion for the treatment of MS in the future (229) (**Figure 2**).

Several therapeutic methods have attempted to target the NF-κB pathway as a critical inflammatory signaling pathway in MФs. The NF-κB family member, c-Rel, is a crucial transcription factor in inflammation and induces pro-inflammatory cytokine production in MФs. Moreover, c-Rel upregulation has been indicated in the spinal cord-infiltrating MФs. Accordingly, Deng et al. (230) have found that silencing of c-Rel in CNS-infiltrating MФs by SiRNA PEG-PLL-PLLeu micelles (cationic micelles based on hybrid polypeptide copolymers [poly (ethylene glycol)-b-poly (L-lysine)-b-poly (L-leucine) (PEG-PLL-PLLeu)] is an effective gene delivery system, which suppresses the clinical signs of EAE and alleviates inflammation in the CNS. Their results showed that these nanoparticles are mainly taken up by F4/80+ cells (CNS-infiltrating inflammatory MФs and microglia). Furthermore, following downregulation of the c-Rel expression in MФs, IFN-γ and IL-17A production by MOG-specific T cells were suppressed in EAE mouse spleen. So, C-Rel targeting in MФs, which dampens Th1 and Th17 responses in EAE, will be helpful for future research on MS treatment (231).

#### Promote Activation, Migration, and Phagocytosis of Myelin Debris

Although autoantibodies are a hallmark of MS disease, natural IgM antibodies usually have beneficial functions in the body (232). rHIgM22 is a human recombinant type of IgM that has been shown to promote remyelination in cuprizone-mediated animal models of MS (233). Zorina et al. (234) have demonstrated that treatment with rHIgM22 increases myelin uptake in microglial cells compared to the Ctrl IgM treatment. CR3 and IgM Fc domain are required for rHIgM22-mediated phagocytosis (235). Therefore, the addition of anti-CD11b antibody (CR3 consists of two subunits, CD11b and CD18) and Fc5μ antibody results in a negative response to rHIgM22. Moreover, in compstatin (C3 inhibitor)-pretreated BV-2 cells, rHIgM22-mediated myelin uptake has wholly blocked. Thus, it seems that complement opsonization is necessary, whereas multiple receptors may be involved (234, 236) (**Figure 1**). Nevertheless, more research will shed light on rHIgM22 functions and their effectiveness in the treatment of MS.

M-CSF is a major cytokine in changing microglial phenotype into an anti-inflammatory subtype. Also, it has many roles in the survival, proliferation, and differentiation of myeloid cells (237, 238). In a study, Laflamme et al. (239) have found that M-CSF administration in the cuprizone EAE mouse model diminishes demyelination and improves myelin sheath overall organization. In addition, M-CSF augments microgliosis (increasing immunoreactivity for Iba-1 indicates microgliosis) and increases the expression of TREM2 mRNA (239) (**Figure 1**).

Ionotropic P2X receptors (P2XRs) are nucleotide-gated ion channels of the P2R family (240). In EAE and human MS, activated microglia highly express Purinergic P2X4R, which makes these receptors remarkable (241).

Ivermectin (IVM) is a semisynthetic macrocyclic lactone that FDA has approved for parasitic disease treatment. IVM interacts with P2X4R and allosterically modulates ion channels (242, 243). Interestingly Zabala et al. (244) have reported that IVM promotes remyelination in the lysolecithin-induced demyelination model in organotypic cerebellar slices. Also, decreased expression of pro-inflammatory genes *vs*. increased anti-inflammatory gene expression has been found during polarization (244). Furthermore, another study has shown that P2X4R locates intracellularly in late endosomes and lysosome membranes (245). Interaction between IVM and P2X4Rs induces lysosome fusion subsequently and leads to acidic endolysosome generation and altogether promotes phagocytic capacity in anti-inflammatory microglia (244) (**Figure 1**).

#### Polarization of Microglia and Macrophages to an Anti-Inflammatory Phenotype by Some Therapeutic Agents

In a study, Yu et al. (246) have presented that msh-like homeobox-3(MSX3) increases M2 polarization and impedes microglia M1 polarization through interfering with MSX3 expression in microglia. In this state, expression of IGF-1, CD206, and FIZZ-1 mRNA levels decreased, but the expression of IL-1β, iNOS, and TNF-α mRNA increased. In contrast, overexpression of MSX3 in microglia has induced a reduction in IL-1β, iNOS, and TNF-α mRNA expression and increased FIZZ-1, CD206, IGF-1, and activin-A mRNA expression. IGF-1 and activin-A are M2-derived factors that promote maturation and survival of oligodendrocyte precursor cells (103, 246–248). Moreover, the overexpression of MSX3 has induced upregulation of peroxisome proliferator-activated receptor (PPAR)γ, JAK3, and STAT6 genes associated with M2 polarization. Interestingly, transplantation of MSX3-overexpressed microglia has improved remyelination and alleviated signs of disease in EAE mice. Also, overexpression of MSX3 in human microglia has shown similar results (246). Based on these results, targeting MSX3 could be assessed as a therapeutic protocol in the future.

In another study, Aryanpour et al. (249) have shown that progesterone therapy increases M2 phenotype-related mRNAs (TREM-2, CD206, Arg-1, and TGF-β) and, in contrast, leads to depletion of M1-microglia markers (iNOS, CD86, MHC-II, and TNF-α) in cuprizone-induced demyelinated mouse model. Moreover, the protein and mRNA expressions of NLRP-3 and IL-18 have been decreased after progesterone therapy. According to a significant decrease in the percentage of demyelination areas after progesterone therapy and its effect on diminishing inflammation (249), future research should consider the potential impacts of this therapy in MS (**Figure 1** **)**.

The monocyte-derived MФs are highly plastic cells, like microglia, which can repolarize to other phenotypes based on exposure to a different condition. Lenalidomide, an oral FDA-approved drug, is used for myelodysplastic syndromes and multiple myeloma treatment (250). Also, its immunosuppressive and neuroprotective effects have been indicated in EAE. Weng et al. (251) have found that lenalidomide ameliorates EAE symptoms from the early stage and lasts until the end of experiment. It also reduces demyelination due to MФ polarization toward M2 phenotype *via* IL10–STAT3–IL10 positive feedback loop. This state leads to IL-10 production and subsequent suppression of pro-inflammatory Th1 and Th17 cell responses. So lenalidomide could be considered as a potential therapeutic drug candidate for attenuating neuronal demyelination in CNS of MS patients (251) (**Figure 2**).

On the other hand, studies have shown that voltage-gated potassium channels 1.3 (Kv1.3) in T cells, microglia, and MФs are necessary for activation, proliferation, and cytokine production of cells (252, 253). Accordingly, Fan et al. (254) have designed an EAE vaccine composed of a B-cell epitope from a pore reign peptide between extracellular loop S5 and S6 on Kv1.3 channels with a universal synthetic T-cell epitope, Pan HLA DR-binding peptide (PADRE). Following the immunization of rats by the PADRE-Kv1.3 vaccine and subsequent induction of EAE, microglia and MФ populations have significantly reduced at the first peak day of the disease. Also, they have shifted to the M2 phenotype with the decrease in iNOS expression and increase in Arg-1. Regarding the protective role of this vaccine in preventing or treating EAE through balancing immune responses, this could be a promising option for MS treatment in the future (254) (**Figure 2**).

Bryostatin-1 (bryo-1) is a macrocyclic lactone that can pass through CNS and affect the immune system. This compound favors an anti-inflammatory environment by inducing a type 2 phenotype (255, 256). Kornberg et al. (257) have administered bryo-1 to EAE mice at the first clinical sign of motor weakness, corresponding to tail paralysis and also on 10 days after peak disease, and observed the promotion of anti-inflammatory phenotype in MФs. So, exploring bryo-1 effects on inflammation in MS might be a promising idea for future research (**Figure 2**).

A natural polyamine, spermidine, is produced from arginine by arginase enzyme (258), and according to the study by Yang et al. (259), administration of spermidine in EAE mice has attenuated disease symptoms and reduced the infiltration of CD4+ T cells and CD11b+ MФs into the CNS. The amelioration by spermidine has relied on shifting MФs phenotype from inflammatory (high expression of CD80 and CD86 and secretion of IL-1β, IL-12, IL-6, and TNF-α) to anti-inflammatory M2 phenotype (downregulation of NF-κB, Il6, Il1b, Il12, as well as Nos2 and upregulation of Arg1). Interestingly, this study’s results have shown that MФs of spermidine-treated mice could transfer the protective effect and alleviate disease severity in EAE. This study has introduced spermidine as a possible drug candidate for MS treatment in the future (259) (**Figure 2**).

Moreover, Veremeyko (260) have demonstrated forskolin’s (coleonol) effects, a plant-derived traditional oriental medicine, on the experimental model of MS. In forskolin-treated EAE mice, downregulation of MHC-I, CD86, and NOS2 on microglia and MФs in the CNS has been observed. In contrast, forskolin treatment has induced upregulation of miR-124, Arg1, Mrc1, Ym1, and Fizz1 on CNS microglia and MФs, leading to polarization anti-inflammatory M2 phenotype. In this state, the changing balance through activating the ERK pathway has decreased neuroinflammation in EAE mice (260).

The other compound, fasudil [1-(5-isoquinolinesulfonyl)-homo-piperazine], with impact on CNS MФs, is a selective Rho kinase (ROCK) inhibitor that inhibits cell migration, proliferation, and survival and used to treat some neural diseases (261, 262). Following fasudil administration by Liu et al. (263), the disease severity has alleviated in early and late treated EAE mice. Besides, MФs have shifted from M1 to M2 phenotype (decrease in M1 markers iNOS, TLR-4, and CD40 expression *vs*. increase in M2 markers CD206 and Arg-1). Furthermore, the level of IL-10 as an anti-inflammatory cytokine has increased after fasudil treatment. This study has suggested further research on the possible role of fasudil in MS treatment (263) (**Figure 2**). In addition to the effects of some drugs or natural compounds on MФs, some cytokines also change CNS MФ phenotype and have potential therapeutic impacts. For example, IL-33 is one of the crucial cytokines of the immune system that can promote Th2-cell expansion and skews MФs toward the M2 activation state (264). In a study, Jiang et al. (265) have presented that treatment of EAE mice with IL-33 facilitates the polarization of alternatively activated MФs and reduces inflammation of the CNS. However, the exact function of IL-33 in the CNS is unclear and needs more investigation in MS therapy (265) (**Figure 2**).

Recent studies have shown that neural stem cell transplantation (NSCT) ameliorates CNS inflammation in animal models by modulating the immune responses (266, 267). Peruzzotti-Jametti et al. (268) have shown that NSCT in EAE mice alleviates disease signs and inflammation by reducing succinate levels in CSF, leading to: 1) a decrease in mononuclear phagocyte (MP) infiltration and 2) secretion of prostaglandin E2 (PGE2), which reprograms type 1 MPs toward an anti-inflammatory phenotype. This study has recommended a new anti-inflammatory mechanism for possible treatment of MS in the future (268) (**Figure 2**).

We know glucocorticoids (GCs) as strong immunosuppressive drugs widely used in treating MS and various inflammatory diseases. GCs can suppress the immune system by many mechanisms like inhibition of cytokine secretion and leukocyte migration, increasing T-cell apoptosis, and shifting MФ polarization (269). It is documented that MФ reaches anti-inflammatory phenotype following exposure to GC, accompanied by the limitation of immune responses and resolution of disease symptoms (270). Montes-Cobos et al. (271) have applied GC *via* inorganic–organic hybrid nanoparticles (IOH-NP) with [ZrO]2+{[betamethasone phosphate (BMP)]0.9[Flavin mononucleotide (FMN)]0.1}2-(BMP-NP). They have found that MФs are polarized to anti-inflammatory phenotype (decreased percentages of MHC class II+ and CD86+ cells) in EAE treated mice. Thus, MФ polarization is crucial for the efficacy of BMP-NP treatment. Based on the potential of BMP-NP as a suitable nanoformulation for GC therapy without toxicity, future investigations should be expanded to examine its potential effects in the treatment of MS and other autoinflammatory diseases (271) (**Figure 2**).

## Conclusion

The role of MФs and microglia in neuroinflammation and MS pathogenesis calls our attention to the use of different therapeutic agents that target these cells. Microglia recognize infections, toxins, and injuries and have a role in maintaining homeostasis in the adult CNS. Activation of microglia also induces the expression of different inflammatory transcription factors such as NF-κB, JAK/STAT, JNK, ERK1/2, and p38. Moreover, different cytokines, including IL-6, IL-8, IL-12, IL-23, IL-1b, and TNF, are produced after microglia activation. The production of chemokines such a CCL2, CCL3, and CCL4 is also induced by activated microglia, which can facilitate leukocyte recruitment in the early phase of MS disease. During the effector stage of EAE, monocytes rapidly infiltrate surrounding meninges, perivascular space, and choroid plexus through and differentiate into MФs. These MФs contribute to the progression of the paralytic stage of EAE and demyelination by expressing MHC-II, costimulatory molecules, and producing pro-inflammatory factors. In EAE, MФ depletion is associated with a lower CNS injury and attenuated signs and symptoms of the disease. So, both resident MФs in the CNS and monocyte-derived MФs that enter into the CNS following alteration in CNS homeostasis play an essential role in neuroinflammation. Moreover, due to their different origin, location, and turnover, other strategies may target various myeloid cell populations. Although the main targets of some drugs in MS treatment are not MФs and microglia cells, they influence these cells indirectly. For example, DMTs, such as IFN-β, fingolimod, and GA, can change the activation, migration, and polarization of M1/M2 MФs and microglia. Also, many therapeutic agents whose impacts on MФs have been assessed *in vitro* or in animal models. Researchers have recently examined various methods of drug delivery by MФs or their products to the CNS. For example, Tong et al. (272) have found monocyte-derived MФs mediate the delivery of superparamagnetic iron oxide nanoparticles (SPIONs, cell-based delivery systems) into the inflamed brain. They have indicated that monocyte-derived MФs uptake SPIONs with different sizes and carry them into the inflamed brain *in vivo* (272) (**Figure 2**). Also, MФ-derived exosomes have been investigated as possible drug delivery agents to the CNS (273). Overall, understanding the exact mechanism of therapeutic agents on MФ population and determining the precise role of MФs as a drug delivery system in CNS will help their usage in clinical studies.

## Author Contributions

MR and PK wrote most parts of manuscript and searched for data and collected information. NE helped in finding information and reviewed the article before submission not only for spelling and grammar but also for its intellectual content and contributed to the design and implementation of the manuscript. All authors discussed the information and commented on the manuscript. All authors contributed to the article and approved the submitted version.

## Conflict of Interest

The authors declare that the research was conducted in the absence of any commercial or financial relationships that could be construed as a potential conflict of interest.

## Publisher’s Note

All claims expressed in this article are solely those of the authors and do not necessarily represent those of their affiliated organizations, or those of the publisher, the editors and the reviewers. Any product that may be evaluated in this article, or claim that may be made by its manufacturer, is not guaranteed or endorsed by the publisher.
